# Bacterial aetiology of chronic otitis media with effusion in children - risk factors

**DOI:** 10.1186/s40463-020-00418-5

**Published:** 2020-04-29

**Authors:** Izabela Korona-Glowniak, Agata Wisniewska, Marek Juda, Karolina Kielbik, Grazyna Niedzielska, Anna Malm

**Affiliations:** 1 Department of Pharmaceutical Microbiology, Medical University of Lublin, 1 W. Chodzki Str, 20-093 Lublin, Poland; 2 Department of Pediatric Otolaryngology, Phoniatrics and Audiology, Medical University of Lublin, 6 Prof. A. Gebali Str, 20-093 Lublin, Poland

**Keywords:** Otitis media with effusion, Otopathogens, Risk factors

## Abstract

**Background:**

Otitis media with effusion (OME) may occur spontaneously because of poor Eustachian tube function or as an inflammatory response following AOM. Bacterial involvement in OME has been widely reported, with various available methods to identify pathogens from middle ear effusion, including traditional culture methods and polymerase chain reaction (PCR).

**Objectives:**

The primary goal of this study was to evaluate the bacteriological profile of middle ear effusion in OME. Risk factors of the bacterial OME aetiology were also identified.

**Methods:**

Middle ear effusions (MEF) from 50 children, aged 2–8 years, diagnosed by ENT and undergoing routine tympanostomy tube placement were collected. MEF samples were streaked on standard microbiological media. Next, DNA was isolated from MEF samples and analysed with multiplex PCR for *Streptococcus pneumoniae, Haemophilus influenzae, Moraxella catarrhalis* and *Alloiococcus otitidis*.

**Results:**

In multiplex PCR assay 37 (74%) of 50 children were positive for at least one of the four microorganisms. In 27.0% positive children multiple bacterial pathogens were identified. *A. otitidis* was the most frequently identified in positive MEF children (59.5%). By multiplex PCR, *H. influenzae, S. pneumoniae* and *M. catarrhalis* were detected in 24, 18 and 8% of OME patients, respectively. There was significant association between bilateral infection and *H. influenzae* aetiology of OME.

**Conclusions:**

Overall we found OME predominantly a single otopathogen infection caused mainly by *A. otitidis*, which is difficult in identification using standard culture method, ahead to *S. pneumoniae* and *H. influenzae*. However, one third of MEF samples had multiple bacterial pathogens.

## Introduction

Otitis media with effusion (OME) is one of the most common otologic diseases of childhood. OME may occur spontaneously, however it can be persistent in some cases, as a result of poor Eustachian tube function or as an inflammatory response following acute otitis media (AOM) [1, 2]. About 80% of the children suffered from this disease by the age of 10 with the highest prevalence at ages of 2 and 5. OME exhibits non-purulent middle ear effusion with the absence of acute infection typical for AOM appearing as middle ear inflammation, including fever and otalgia [1, 2]. It has been postulated that the Eustachian tube short length, horizontal position, and reduced rigidity in the pediatric population may permit the reflux of naso- and oropharyngeal microbes into the middle ear cavity, explaining the higher incidence of OME in children compared to adults [1].

Bacterial involvement in OME has been widely reported, with various available methods to identify pathogens from middle ear effusion, including traditional culture methods and polymerase chain reaction (PCR). Traditional culture-based techniques have isolated *Streptococcus pneumoniae, Haemophilus influenzae, Moraxella catarrhalis* and *Staphylococcus epidermidis* as the most common pathogens in OME [2]. However, in chronic OME (duration < 3 months), middle ear fluid (MEF) cultures yield positive results for only 20–30% of patients [2]. Polymerase chain reaction (PCR) and 16S rRNA sequencing-based methods identified different species as a possible pathogens in the development of OME. One such emerging potential pathogen is *Alloiococcus otitidis*, which has been isolated from middle ear aspirates with increasing frequency in the last decade [3–5]. Therefore, the primary goal of this study was to evaluate the bacteriological profile of middle ear effusion in OME with use of traditional culture method and PCR technique. This is the first study identifying the risk factors of the infection by particular otopathogens in OME.

## Materials and methods

### Patients

This prospective study enrolled 50 children, aged between 2 and 8 years who were diagnosed with OME during 2017–2018. All children with preliminary OME diagnosis by paediatricians as well as children directly diagnosed by Ear-Nose-and-Throat (ENT) specialists, were assessed for eligibility. Eligible children, once the OME diagnosis was confirmed by the ENT specialists in the Department of Pediatric Otolaryngology, Phoniatrics and Audiology, Medical University of Lublin, Poland, were enrolled to the study. The criteria for surgery were the presence of middle ear effusion for more than 3 months and not receiving antibiotic therapy for at least 2 weeks prior to the procedure. Children with tympanic membrane perforations, immunological defects, any malformations, respiratory tract infection and purulent middle ear fluid were excluded.

A total of 68 specimens of middle ear effusions (MEF) were obtained from 50 children (17 females and 33 males, from 2 to 8 years, median age of 4 years). In 19 out of 50 children with bilateral OME, two separate specimens were taken from each ear at the same episode. The rest of the children were diagnosed with unilateral OME. The pathogens which were detected from both ears were double counted. However, for statistical analysis of risk factors associated with OME pathogens were accounted once in a single patient if detected at least in one ear.

Diagnosis of OME was based on video-otoscopy (yellow or amber color, decreased mobility, air fluid level and retracted tympanic membrane), type B or C tympanometric curves and absence of otoacoustic emissions. Children between 5 and 8 years old also underwent pure tone audiometry to evaluate the level of hearing loss. An original questionnaire was developed based on the state-of-the-art literature. The questionnaire included: demographic data (age, sex, residency, social status in personal evaluation), parental smoking, type of delivery, breastfeeding and health status: all types of allergies, number of upper respiratory tract infections per year (more than 6 per year was reported as frequent), duration of the otitis media symptoms, snoring and history of pneumococcal vaccination.

### Study procedures

All of the specimens were obtained during myringotomy, performed as the treatment for OME. Antisepsis of the ear canal before the procedure was performed by installing Octenisept (Schülke&Mayr) for 1 min. After removing it by suction, myringotomy was carried out, during which dense, mucopurulent discharge draining under pressure from middle ear was found uni- or bilaterally. MEF sample was collected from the ear with the use of sterile suction needle inserted into the middle ear cavity through the incision in the tympanic membrane. The samples were placed in sterile eppendorf tubes and, maintained in 37 °C, transported immediately to the laboratory. The remaining amount of exudate had been aspirated. Next, in 29 (46.8%) patients ventilation drainage of the middle ear had been placed.

### Laboratory procedures

MEF samples were inoculated on Mueller-Hinton agar with 5% sheep blood, Mueller-Hinton agar with 5% sheep blood with 0.5 mg/L of gentamicin for selective cultivation of pneumococci, Haemophilus chocolate agar (BioMerieux) for selective cultivation of *Haemophilus* sp. and Chapman agar for selective cultivation of staphylococci. Plates were incubated for 24–48 h at 35 °C under aerobic conditions or in 5% CO_2_ enriched atmosphere. Pneumococci were identified by colony morphology, susceptibility to optochin (5 μg, BioMereieux), and bile solubility; identification was confirmed by a slide agglutination test (Slidex Pneumo-Kit, BioMerieux). *S. pyogenes* was identified by colony morphology, susceptibility to bacitracin (Bacitracin disk, 0.04 U, Sigma-Aldrich) and confirmed by slide agglutination test Slidex Strepto plus (BioMerieux). *M. catarrhalis* and *H. influenzae* were identified by macroscopic, microscopic and biochemical assays by API NH microtest (BioMerieux). Isolates of *S. aureus* were identified by colony morphology, biochemical activities (ID32 STAPH, BioMerieux), coagulase test and a slide agglutination test (Slidex Staph-Kit, BioMerieux).

Next, DNA from MEF samples were extracted using QIAGEN QIAamp DNA Mini Kit (Qiagen, USA) according to the manufacturer’s instructions and analyzed with multiplex PCR with the use of method described elsewhere [5]. The PCR mixture contained the following primers for *A. otitis* (3.6 μM), *H. influenzae* (1.8 μM), *M. catarrhalis* (0.6 μM) and *S. pneumoniae* (0.2 μM) as well as common reverse primer (0.8 μM). Moreover the concentration of MgCl_2_ was 2 mM, 3 units of *Taq* polymerase (Thermo Scientific) were used per reaction. The reaction profile was 3 min of initial denaturation and 35 cycles of 94 °C for 30 s, 66 °C for 1.5 min and 72 °C for 1 min followed by a 5 min final extension at 72 °C. The PCR products were separated in 2% agarose gen in Tris-borate-EDTA buffer and DNA bands were visualized with SimplySafe dye (Euryx) by UV light illumination.

### Statistical analysis

Data processing and analysis were performed using Tibco Statistica Ver. 13.3 (TibcoSoft. Inc.). The results are expressed as percentage or median with range. Univariate analyses were performed using Chi-square or Fisher exact test, depending on size of samples and of contingency tables for categorical variables and using Mann-Whitney U test for continuous variables. Odds ratios (OR) and their 95% confidence intervals (CI) were calculated. Statistical significance was set if the 2-tailed *p* value was < 0.05.

## Results

Of the 68 MEF specimens, positive culture was observed in 6 (8.8%) specimens. By PCR, altogether 50 (73.5%) specimens were positive for one of the four otopathogens. Of these 50 positive samples, 12 (24%) had 2–3 bacterial species detected. By combination of culture and PCR, 50 (73.5%) of the 68 OME specimens were positive for bacterial pathogens. Comparing the results of otopathogen isolation by microbiological cultures and PCR tests, the sensitivity and negative predictive values for culture method were very low and amounted to 12.0% (95%CI 0.05–0.24) and 29.0% (95%CI 0.18–0.42), respectively (Table S1). As shown in Table 1 by culture and PCR, *A. otitidis* was the most frequent pathogen in OME specimens.

**Table 1:** Frequency of *otopathogens* in 68 samples of middle ear effusions from otitis media patients

Pathogen	No. (%) of positive specimens		
	Culture^a^	PCR	Total
*A. otitidis*	0 (0)	30 (44.1)	30 (44.1)
*S. pneumoniae*	3 (4,4)	9 (13,2)	10 (14.7)
*H. influenzae*	0 (0)	14 (20.6)	14 (20.6)
*M. catarrhalis*	0 (0)	6 (8.8)	6 (8.8)

^a^The other pathogens which were detected by culture were *Staphylococcus aureus* in 2 (2.9%) samples and Coagulase-negative staphylococcus in 1 (1.5%) sample

Positive culture results were reported for only 6 (10%) children - *S. pneumoniae* (3 patients), *S. aureus* (2 patients) and CNS (1 patient). In multiplex PCR assay, 37 (74%) of 50 children were positive for at least one of the four microorganisms. In 27.0% of positive children multiple bacterial pathogens were identified. *A. otitidis* was the most frequently identified in positive MEF children (59.5%) (Table 2). As a single otopathogen, it was observed in 15 positive children (40.5%). *S. pneumoniae* was identified in 9 (24.3%) positive children - in 13.5% as a single otopathogen. *H. influenzae* (33.4% of positive children) was more frequently identified with the other pathogens (in 7 out of 12 children). Only 4 (10.8%) of positive MEF children had *M. catarrhalis* identified.

**Table 2:** Characteristics of children with OME

Characteristics	Total (*n* = 50)	MEF positive (% in group)			
		*S. pneumoniae* (*n* = 9)	*H. influenzae* (*n* = 12)	*A. otitidis* (*n* = 22)	*M. catarrhalis* (*n* = 4)
Age (yr)^a^	4 (2–8)	5 (3–8)	4 (2–7)	3.7 (3–5)	4 (3–6)
Male sex	33 (66.0)	8 (24.2)	6 (18.2)	13 (39.4)	3 (9.1)
Social status:					
Very good	26 (52.0)	2 (7.7)	6 (23.1)	11 (42.3)	1 (3.9)
Good	23 (46..0)	7 (30.4)	5 (21.7)	10 (43.5)	3 (13.0)
Low	1 (2.0)	0 (0)	1 (100)	1 (100)	0 (0)
Rural residence	19 (38.0)	4 (21.1)	4 (21.1)	10 (52.6)	3 (15.8)
Parental smoking	14 (28.0)	4 (28.6)	6 (42.9)	8 (57.1)	2 (14.3)
Breastfeeding	45 (90.0)	9 (20.0)	8 (17.8)	19 (42.2)	4 (8.9)
Cesarean childbirth	22 (44.0)	5 (55.6)	4 (18.2)	11 (50.0)	2 (9.1)
Bilateral OME: positive samples from right/left ear	19 (38.0): 15/13	1 (5.3): 1/0	9 (47.4): 8/6	8 (42.1): 8/8	3 (15.8): 3/2
Unilateral OME	31 (62.0)	8 (25.8)	3 (9.7)	14 (45.2)	1 (3.2)
Duration of symptoms (days)^a^	639 (92–1095)	548 (365–730)	548 (183–730)	730 (183–1095)	730 (365–730)
Frequent URTI	39 (78.0)	8 (20.5)	9 (23.1)	15 (38.5)	4 (10.3)
Snoring	34 (68.0)	4 (11.8)	8 (23.5)	16 (47.1)	3 (8.8)
Allergy	7 (14.0)	1 (14.3)	1 (14.3)	1 (14.3)	1 (14.3)
Pneumococcal vaccination	16 (32.0)	4 (25.0)	5 (31.3)	5 (31.3)	1 (6.3)

^a^median (range)

**Fig. 1:**
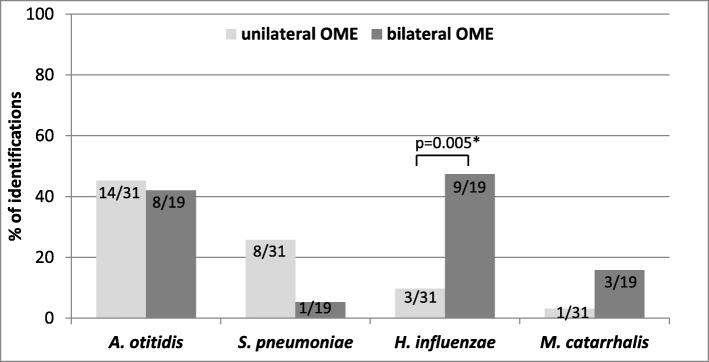
Prevalence of otopathogens in MEF samples in uni- and bilateral OME with use PCR method. Number of identifications in relation to 31 of unilateral cases and 19 of bilateral cases is presented in each bar

Among 19 patients with bilateral OME the most frequent otopathogen was *H. influenzae* (9/19 patients; 47.4%). The higher prevalence of this otopathogen in bilateral infection in comparison to unilaterally affected patients was statistically significant (RR 4.9, 95%CI 1.5–15.8, *p* = 0.005). *S. pneumoniae* was mainly shown as the cause of unilateral OME (8/31 patients; 25.8%) but without statistical significance (RR 4.9, 95%CI 0.7–36.2, *p* = 0.13) (Fig. 1). When analyzing bilateral samples, all patients with *A. otitidis* were infected in both ears (8/8 patients). Five out of 9 bilaterally affected patients with *H. influenzae* were infected in both ears and 1 out of 3 patients with *M. catarrhalis* was infected in both ears. *S. pneumoniae* was present in only one bilaterally affected patient, but this otopathogen was detected in only one ear (Table 2).

Demographic and clinical data of studied children were shown in Table 2 together with description of positive children with four tested otopathogens. Statistical analysis indicated *H. influenzae* aetiology as a factor significantly associated with bilateral OME in children. There was a non-statistically significant association (*p* < 0.1) between the presence of bilateral OME and younger age, as well as rural residents (Table 3). Analysis of affecting factors for positive MEF samples in OME patients was determined for *S. pneumoniae, H. influenzae, A. otitidis* and *M. catarrhalis* separately. *H. influenzae* as the otopathogen present in MEF specimens was significantly associated with bilateral OME but negatively with breast feeding in OME patients (Table 4). The univariate analysis did not indicate any factors associated with *M. catarrhalis* and *A. otitidis* presence in MEF of OME patients.

**Table 3:** Analysis of risk factors for bilateral OME in children

Characteristics	OR (95%CI)	*p* value
Age (yr)	0.6 (04–1.0)	0.072
Male sex	1.2 (0.4–4.0)	0.78
Good social status	0.9 (0.3–2.7)	0.79
Rural residence	2.7 (0.8–8.9)	0.099
Parental smoking	0.9 (0.2–3.1)	0.84
Breastfeeding	0.4 (0.06–2.4)	0.30
Cesarean childbirth	0.9 (0.3–2.8)	0.83
*S. pneumoniae* positive	0.2 (0.02–1.4)	0.097
*H. influenzae* positive	8.4 (1.9–37.4)	0.005
Duration of symptoms	1.0 (0.99–1.004)	0.21
Frequent URTI^a^	0.4 (0.1–1.6)	0.21
Snoring	0.7 (0.2–2.4)	0.57
Allergy	1.3 (0.3–6.4)	0.78
Pneumococcal vaccination	1.4 (0.4–4.8)	0.57

^a^Upper respiratory tract infections

**Table 4:** The associations of epidemiological factors with *Streptococcus pneumoniae* and *Haemophilus influenzae* MEF positive samples in children with OME

Characteristics	*S. pneumoniae*		*H. influenzae*	
	OR (95%CI)	*p* value	OR (95%CI)	*p* value
Age (yr)	1.7 (0.9–3.0)	0.088	0.9 (0.5–1.5)	0.60
Male sex	5.1 (0.6–44.9)	0.14	0.4 (0.1–1.5)	0.19
Good social status	5.3 (1.0–28.6)	0.055	0.9 (0.2–3.6)	0.91
Rural residence	1.4 (0.3–6.0)	0.66	0.8 (0.2–3.0)	0.70
Parental smoking	2.5 (0.6–11.1)	0.23	3.8 (0.9–14.8)	0.06
Breastfeeding	–	–	0.05 (0.005–0.6)	0.014
Cesarean childbirth	1.8 (0.4–7.6)	0.44	0.6 (0.1–2.2)	0.40
Bilateral OME	0.16 (0.02–1.4)	0.097	8.4 (1.9–37.4)	0.005
Duration of symptoms	1.0 (0.99–1.0)	0.89	1.0 (0.99–1.0)	0.46
Frequent URTI	2.6 (0.3–23.2)	0.40	0.8 (0.2–3.7)	0.77
Snoring	0.3 (0.07–1.3)	0.11	0.9 (0.2–3.7)	0.91
Allergy	0.7 (0.08–6.9)	0.78	0.5 (0.05–4.5)	0.52
Pneumococcal vaccination	1.9 (0.4–8.5)	0.38	1.8 (0.5–6.7)	0.41

## Discussion

In this study, we have determined bacterial aetiology of OME both by bacterial culture isolation and PCR detection of four otopathogens in children undergoing myringotomy. Standard bacterial culture and sensitive molecular detection techniques have shown that the healthy middle ear is typically a sterile site [6]. Similarly to others, multiplex-PCR method had a better performance than conventional culture method in detecting middle ear pathogens in the MEFs samples (74% versus 10%, respectively). Low sensitivity (12%) of conventional culture methods, their time-consumption, labor-intensiveness and need of experience in microbiological diagnosis convince that multiplex-PCR, being a one-step rapid method, might be considered as a routine diagnostic method in patients with OME [3, 7, 8]. *A. otitidis* was the most frequent otopathogen (44%) in both unilaterally (42%) and bilaterally (46.7%) affected patients, though without statistical significance. This pathogen was not identified in the course of culture method. By multiplex PCR, *H. influenzae, S. pneumoniae* and *M. catarrhalis* were detected in 24, 18 and 8% of OME patients, respectively, similarly to other reports [3, 8, 9].

Bacterial isolation rates from patients with AOM have been found to range from 50 to 90%, but to be lower (21 to 70%) in patients with OME [7, 9, 10]. The role of bacteria in aetiology of OME has been controversial. OME is not characterized by symptoms and signs of acute inflammation that would be expected in a typical acute bacterial infection caused by planktonic bacteria. The typical bacteria involved in OME are *S. pneumoniae*, *M. catarrhalis* and *H. influenzae*, but in most studies bacteria were cultured in less than half of the samples, ranging from 21 to 70% [7, 11, 12]. In our study, positive bacterial cultures were even less (10%). Although this may suggest that bacteria are not important in OME, it contradicts studies examining MEF for the presence of bacterial nucleic acids by PCR, which have demonstrated bacterial DNA typically in excess of 80% of effusions [6, 13]. However, the presence of bacterial nucleic acids does not necessarily equate to the presence of viable bacteria as components of effusion samples have been shown to inhibit nuclease activity, and this has been postulated to cause the persistence of RNA and DNA even if bacteria are no longer viable [14]. However, Stoodley et al. [15] using confocal microscopy found that 92% of a population of children presenting OME had live bacteria (*S. pneumoniae*, *H. influenza*, and *M. catarrhalis*) in their mucosal biopsies. These metabolically active bacteria might be present in at least half of all cases of OME with negative bacterial cultures and are thought to participate in biofilm formation [16]. Moreover, Daniel et al. [17] reported that 45.2% of MEF samples in OME were culture-positive, but 82.3% were positive by confocal laser scanning microscopy and bacteria viability stain. Combining the two techniques they demonstrated live bacteria in 91.8% of samples. Rayner et al. [18] have demonstrated the presence of *H. influenzae* mRNA using reverse transcriptase PCR in 43% of 93 middle ear effusions from children with OME for 3 months or more, when only 12% were positive on culture. The authors argue that bacterial mRNAs have a half-life of seconds, so their presence indicates viable and metabolically active bacteria. Biofilm formation may be a causative factor in culture-negative OME [15, 16, 18] since it is a state of very low metabolic activity, almost a suspended animation and bacteria in this state are resistant to antibiotics, but may still elicit an immune response, which will result in the production of the mucin-rich effusion.

Indeed, even though *A. otitidis* has been identified by molecular methods in significant proportions of children with acute otitis media (AOM), it is not often isolated from clinical material since the incubation period required for its detection is longer than those used in routine diagnostic laboratories [19, 20]. The role of *A. otitidis* in pathogenesis of OM is unclear. However, a small number of studies has reported findings that support the hypothesis of *A. otitidis* being a pathogen. The bacteria activated lymphocytes and induced production of pro-inflammatory cytokines in similar amounts as the major middle ear pathogens [21–23].

Considering that *H. influenzae*, *S*. *pneumoniae* and *M*. *catarrhalis* are the most frequent pathogens detected in acute otitis media and are able to ascend through the Eustachian tube, causing ciliary damage to the airway epithelium and disrupting mucociliary flow, this may result in conditions for persistence in the middle ear compartment [9, 10, 24, 25]. In some cases, especially when OME is not clearly related to a mechanical obstruction of the Eustachian tube, the Toynbee effect of negative pressure within the middle ear may help the ascendance of microbes through the tube, leading to colonization of the middle ear, which may be pivotal in OME pathogenesis [26].

The most interesting finding in our study was the significant association between bilateral infection and *H. influenzae* aetiology of OME, as well as tendency of association between unilateral OME and *S. pneumoniae* aetiology. Various social factors have been implicated in the aetiology of OME, most of which are related to socio-economic class, and all of which are probably mediated by an increased propensity to infection. Studies have shown that children who attend day-care centers have a two- to three-fold increased risk of OME. Also greater exposure to respiratory pathogens, both bacterial and viral, and a seasonal variation with the winter preponderance are the reasons for the increase in the incidence of OME [13]. Breastfeeding exerts a protective effect, perhaps by the effect of maternal antibodies on middle ear pathogens [13]. In our study we have made the first attempt to find factors associated with specific bacterial aetiology of OME. Protective effect of breastfeeding along with association with bilateral infection were observed for *H. influenzae* aetiology. There are studies reporting associations between allergy, URTIs, parental smoking, snoring, low social status and chronic/recurrent otitis media, however to our best knowledge none of them determined risk factors associated with particular bacterial aetiology [27].

## Conclusion

Overall we found OME predominantly a single otopathogen infection caused mainly by *A. otitidis*, which is difficult in identification using standard culture method, ahead to *S. pneumoniae* and *H. influenzae*. However, one third of MEF samples had multiple bacterial pathogens. Definitively, PCR was the more sensitive method for identification of OME aetiology in MEF samples. We have demonstrated significant association between bilateral infection and *H. influenzae* aetiology of OME.

## Supplementary information

**Additional file 1: Table S1.** The analysis of differences in sensitivity, specificity and positive and negative predictive values of PCR and culture methods in otopathogens identification.

## Data Availability

The datasets analyzed during the current study are available from the corresponding author on reasonable request.
