# Supplementary material for: Bacterial aetiology of chronic otitis media with effusion in children - risk factors
Source: J Otolaryngol Head Neck Surg. 2020 Apr 29;49:24. doi: 10.1186/s40463-020-00418-5 (PMC7191732; doi:10.1186/s40463-020-00418-5)
Supplement: Supplementary file 1 — Additional file 1: Table S1. The analysis of differences in sensitivity, specificity and positive and negative predictive values of PCR and culture methods in otopathogens identification. [file 40463_2020_418_MOESM1_ESM.docx]

Table S1. The analysis of differences in sensitivity, specificity and positive and negative predictive values of PCR and culture methods in otopathogens identification.

|  |  | **PCR method** | |  |  |
| --- | --- | --- | --- | --- | --- |
| **Culture method** |  | Condition positive | Condition negative |  | 95%CI |
|  | Test positive | 6 | 0 | 1.0  PPV | 0.54-1.0 |
|  | Test negative | 44 | 18 | 0.29  NPV | 0.18-0.42 |
|  |  | Sensitivity  0.12 | Specificity  1.0 |  |  |
|  | 95%CI | 0.045-0.24 | 0.81-1.0 |  |  |
